# Cyclic AMP Effectors in African Trypanosomes Revealed by Genome-Scale RNA Interference Library Screening for Resistance to the Phosphodiesterase Inhibitor CpdA

**DOI:** 10.1128/AAC.00508-13

**Published:** 2013-10

**Authors:** Matthew K. Gould, Sabine Bachmaier, Juma A. M. Ali, Sam Alsford, Daniel N. A. Tagoe, Jane C. Munday, Achim C. Schnaufer, David Horn, Michael Boshart, Harry P. de Koning

**Affiliations:** Institute of Infection, Immunity & Inflammation, College of Medical, Veterinary & Life Sciences, University of Glasgow, Glasgow, United Kingdoma; Biocenter, Section Genetics, Ludwig-Maximilians-Universität München, Martinsried, Germanyb; Faculty of Infectious & Tropical Diseases, London School of Hygiene & Tropical Medicine, London, United Kingdomc; Centre for Immunity, Infection & Evolution, Institute of Immunology & Infection Research, University of Edinburgh, Edinburgh, United Kingdomd; Wellcome Trust Centre for Molecular Parasitology, University of Glasgow, Glasgow, United Kingdome

## Abstract

One of the most promising new targets for trypanocidal drugs to emerge in recent years is the cyclic AMP (cAMP) phosphodiesterase (PDE) activity encoded by *TbrPDEB1* and *TbrPDEB2*. These genes were genetically confirmed as essential, and a high-affinity inhibitor, CpdA, displays potent antitrypanosomal activity. To identify effectors of the elevated cAMP levels resulting from CpdA action and, consequently, potential sites for adaptations giving resistance to PDE inhibitors, resistance to the drug was induced. Selection of mutagenized trypanosomes resulted in resistance to CpdA as well as cross-resistance to membrane-permeable cAMP analogues but not to currently used trypanocidal drugs. Resistance was not due to changes in cAMP levels or in *PDEB* genes. A second approach, a genome-wide RNA interference (RNAi) library screen, returned four genes giving resistance to CpdA upon knockdown. Validation by independent RNAi strategies confirmed resistance to CpdA and suggested a role for the identified cAMP Response Proteins (CARPs) in cAMP action. CARP1 is unique to kinetoplastid parasites and has predicted cyclic nucleotide binding-like domains, and RNAi repression resulted in >100-fold resistance. CARP2 and CARP4 are hypothetical conserved proteins associated with the eukaryotic flagellar proteome or with flagellar function, with an orthologue of CARP4 implicated in human disease. CARP3 is a hypothetical protein, unique to Trypanosoma. CARP1 to CARP4 likely represent components of a novel cAMP signaling pathway in the parasite. As cAMP metabolism is validated as a drug target in Trypanosoma brucei, cAMP effectors highly divergent from the mammalian host, such as *CARP1*, lend themselves to further pharmacological development.

## INTRODUCTION

Human African trypanosomiasis (HAT, or sleeping sickness) is a potentially lethal parasitic disease caused by two subspecies of Trypanosoma brucei, T. brucei rhodesiense and T. brucei gambiense, which have distinct geographical distributions (1). A third subspecies, T. brucei brucei, is noninfective to humans but, alongside Trypanosoma vivax and Trypanosoma congolense, causes huge economic damage through the infection of domestic animals such as cattle, causing a disease known as Nagana or animal African trypanosomiasis (AAT) (2). T. brucei is transmitted to its mammalian hosts via the mouthparts of infected blood-sucking tsetse flies (3). Millions of people in sub-Saharan Africa are at risk of this infection, with over 175,000 cases reported between the years 2000 and 2009 across 25 countries (4); with an estimated 3-fold underreporting (5), as many as half a million people could actually have been infected. In the early stages of the infection (stage I), the trypanosomes proliferate in the peripheral bloodstream and lymph, causing a relatively mild disease of intermittent fever and general malaise, but the penetration of the parasite into the central nervous system (stage II) causes severe neurological symptoms followed by coma and, almost invariably, death (6).

The treatment for stage I HAT is pentamidine for T. brucei gambiense infection and suramin for T. brucei rhodesiense, but since these compounds have at best minimal capacity to cross the blood-brain barrier, they are not suitable for treatment of the second stage of infection (7). Chemotherapies available for stage II HAT are melarsoprol or eflornithine. Melarsoprol is a drug based on arsenic and can have very severe side effects, with up to 5% of patients dying from reactive encephalopathy due to the drug (6). On top of the potential toxicity, resistance to melarsoprol appears to be increasing, with treatment failure rates as high as 37% in some regions (8). Current models describe the loss of one or more transporters, including the TbAT1/P2 adenosine transporter (9), the high-affinity pentamidine transporter (HAPT) (10), and the aquaporin TbAQP2 (11, 12) as being involved in pentamidine/melarsoprol cross-resistance. Eflornithine is effective only against T. brucei gambiense infections and is difficult to administer, requiring hospitalization and intravenous infusions every 6 h for 2 weeks (7), although a recently introduced combination therapy of nifurtimox and eflornithine (NECT) has reduced the treatment burden (13). However, NECT is still not effective against T. brucei rhodesiense, and the need for more-effective drugs with fewer side effects and no cross-resistance is clearly urgent.

From mammals to protozoa and prokaryotes, cyclic AMP (cAMP) generated by adenylate cyclases is an intracellular second messenger in cell signaling. The increase in cAMP concentration transduces the initial stimulus down the signaling cascade by activating or deactivating effector proteins, such as kinases. In T. brucei brucei, a crucial role of cyclase activity, encoded by the most abundant *ESAG4* gene product and probably other members of the large family of adenylate cyclases, is to produce extracellular cAMP as part of the parasite's ability to subvert the host innate immunity upon infection (14).

The impact of changes in intracellular cAMP concentration on trypanosomes is evidenced by the severe phenotypes upon altered expression of enzymes involved in cAMP metabolism. Elevated cAMP is degraded to AMP by phosphodiesterases (PDEs) (15), of which there appear to be four distinct families in T. brucei brucei (16, 17). Recently, cAMP-specific PDEs have been validated genetically and pharmacologically as excellent drug targets in the parasite (18–20). The combined activity of the two members of the *PDEB* family was shown to be essential in bloodstream form trypanosomes. Simultaneous RNA interference (RNAi) knockdown of both *PDEB* genes in T. brucei brucei bloodstream forms generated an uncontrolled and sustained increase in cAMP concentration, resulting in cytokinesis defects producing multinuclear and multiflagellated cells that eventually die (18). A similar impaired-cytokinesis phenotype is produced by repression of adenylate cyclase activity (21). This apparent paradox suggests that fine-tuning of cAMP levels plays a role in regulation of cell division, with extreme or deregulated concentrations in either direction being detrimental (see discussion in reference 21).

A phenotype similar to *PDEB* RNAi is also observed when bloodstream form trypanosomes are exposed to CpdA, a compound that inhibits both TbrPDEB proteins with nanomolar affinity (19). Ongoing drug development work is exploiting unique structural differences between the trypanosomal PDEBs and the equivalent human PDEs in order to increase selectivity (22). The characterization of the first TbrPDE inhibitors also provided the first pharmacological tool to specifically manipulate cAMP levels in kinetoplastid parasites and potentially identify downstream effectors. One promising approach to identify pathways involved in a drug's action is to study drug resistance mechanisms.

In this study, two parallel approaches were used to identify possible modes of resistance to the TbrPDEB inhibitor CpdA. The first attempted to generate resistance by gradually increasing concentrations of the compound in chemically mutagenized bloodstream form cultures, followed by characterization of the surviving cell lines. The second exploited a whole-genome RNA interference screen for genes that confer resistance to CpdA when knocked down. Four candidate genes were identified that were necessary to mediate the lethal drug action of PDE inhibitors and consequently are associated with reduced CpdA sensitivity when knocked down by RNAi. This represents an important advance, as downstream effector proteins of cAMP signaling have not been previously characterized in trypanosomes. We propose that the newly identified genes required for CpdA sensitivity encode the first *bona fide* cAMP effector proteins identified in T. brucei brucei.

## MATERIALS AND METHODS

### Trypanosome strains and culturing.

Bloodstream forms of Trypanosoma brucei brucei strain Lister 427 were grown at 37°C in a 5% CO_2_ atmosphere in HMI-9 medium (23) supplemented with 10% fetal bovine serum (FBS). The CpdA-resistant R0.8 line was derived from wild-type T. brucei brucei strain Lister 427 and cultured under the same conditions as the wild type except that 0.4 μM CpdA was added to the medium to maintain drug pressure. Before assaying, R0.8 trypanosomes were grown in medium without CpdA for at least 6 days (approximately 18 generations). The RNAi cell lines based on MiTat 1.2 13-90 (24) were kept under selection with 2.5 μg/ml Geneticin, 5 μg/ml hygromycin, and 1 μg/ml phleomycin; 0.1 μg/ml puromycin was added to the RNAi cell lines bearing a tagged *CARP* allele.

### RNAi construct generation and transfection.

RNAi fragments were amplified from genomic DNA of T. brucei brucei strain Lister 427 and cloned into the p2T7-177-BLE vector (25) via BamHI and HindIII (or XhoI in the case of *CARP1*) restriction sites. The RNAi target regions were chosen as follows: Tb427tmp.01.7890, 541 bp, positions 1254 to 1794; Tb427tmp.52.0004, 383 bp, positions 528 to 910; Tb427.07.5340, 422 bp, positions 781 to 1202; Tb927.3.1040/60 (the TREU927 reference strain was used here, since the respective sequence in strain Lister 427 is not fully sequenced), 755 bp, positions 835 (Tb927.3.1040) to 395 (Tb927.3.1060) (see Fig. 4 for schematic representations of targeting regions). Primer sequences are available upon request. Electroporation and selection procedures were performed as described previously (26).

### Tagging of CARP proteins.

*In situ* tagging of *CARP1*, *CARP3*, and *CARP4* was performed on pMOTag vectors using a PCR-based strategy (27). CARP1 and CARP4 were fused to a C-terminal 3×HA (hemagglutinin) tag, and CARP3 was fused to a single Ty1 tag using the vectors pMOTag2H or pMOTag2T, respectively (derivatives of the pMOTag2 vector series with puromycin resistance cassette [27]). Primers were designed according to the published protocol with stretches of 60 to 80 nucleotides homologous to the 3′ end of the open reading frame (ORF) or the beginning of the 3′ untranslated region (UTR), respectively. CARP1 was independently tagged with a 4×Ty1 tag at the N terminus using the vector p3077 (derivative of pN-PTP [28]; kindly provided by S. Kramer, Würzburg). An N-terminal fragment of the *CARP1* ORF (positions 1 to 780) was cloned into the vector p3077 via HindIII and EcoRV restriction sites. The construct was linearized with SwaI for transfection. Tagging of CARP2 was based on the vector p3074 (derivative of pC-PTP [28]; kindly provided by S. Kramer, Würzburg) fusing a 4×Ty1 tag to the C terminus of the protein. The *CARP2* ORF was cloned into the vector p3074 via BamHI and SwaI restriction sites. After exchange of the resistance cassette from neomycin to puromycin via BstBI and NdeI restriction sites, the construct was linearized with XhoI for transfection. All primer sequences are available upon request.

### Test compounds.

CpdA and CpdB were synthesized and generously provided by Geert-Jan Sterk, Mercachem, Netherlands. Dipyridamole, etazolate, dibutyryl cAMP, 8-bromo-cAMP, 8-(4-cholorophenylthio)-cAMP (8-CPT-cAMP), pentamidine, phenylarsine oxide, and diminazene were obtained from Sigma-Aldrich and Fluka; melarsen oxide was a gift from Sanofi-Aventis; suramin was a gift from Brian Cover (University of Kent at Canterbury); nifurtimox and eflornithine were gifts from Mike Barrett (University of Glasgow); cymelarsan was a gift from Mike Turner (University of Glasgow). Stock solutions of all compounds were made up in dimethyl sulfoxide (DMSO), with the solvent never exceeding 0.5% (vol/vol) under experimental conditions.

### Induction of resistance to CpdA.

Methyl methanesulfonate (MMS; Sigma) was added to a 50-ml culture of T. brucei brucei strain Lister 427 wild-type trypanosomes in late logarithmic growth phase to give a final concentration of 0.001% (vol/vol), and the mixture was incubated at 37°C and 5% CO_2_ for 1 h. Subsequently, the culture was centrifuged at room temperature (610 × *g*, 10 min) and the supernatant carefully removed and discarded in 1 M NaOH (to deactivate the mutagen). The cell pellet was resuspended in fresh medium and washed twice by centrifugation as above. After the final wash, the pellet was resuspended in 50 ml medium and incubated at 37°C, 5% CO_2_. During this incubation, approximately 95% of the trypanosomes died due to exposure to MMS. The remaining trypanosomes, some of which will have been mutagenized, proliferated. Once the surviving culture reached the late logarithmic phase of growth, the cells were washed once, as above, and resuspended in fresh medium containing 0.1 μM CpdA, at a cell density of 2.5 × 10^4^ cells/ml. The mutagenized trypanosomes were added to multiple 24-well plates and incubated at 37°C, 5% CO_2_. Cell viability was checked by light microscopy every 24 h for 5 days. Once the trypanosomes in a well reached the late logarithmic phase of growth, they were passaged into 3 wells of a new 24-well plate with fresh medium: one containing CpdA at the screening concentration, another at 2× the screening concentration, and the third being a no-drug control. The cultures were thus continuously maintained under gradually increasing (doubling), sublethal concentrations of CpdA.

### Dose-response cell viability assay.

The efficacies of test compounds against various cell lines of T. brucei brucei strain Lister 427 were determined using a modified version of the alamarBlue assay described previously (29, 30). Briefly, test compounds were doubly diluted in white-bottomed 96-well plates (Greiner) with standard culture medium. An equal volume (100 μl) of bloodstream form trypanosomes in medium was added to each well to give a final cell density of 1 × 10^5^ trypanosomes/ml. The plates were incubated for 48 h at 37°C, 5% CO_2_, after which 20 μl of 0.5 mM resazurin sodium salt (Sigma) in phosphate-buffered saline (PBS) was added to each well, followed by a further 24-hour incubation under the same conditions. RNAi lines were induced with 1 μg/ml tetracycline (Tet; Sigma) 24 h prior to plating in test compound dilutions, and Tet was included until the end of the experiment.

Following the final incubation, fluorescence was measured using a FLUOstar Optima fluorimeter (BMG Labtech) with excitation and emission filters of 544 nm and 590 nm, respectively. Data were analyzed using GraphPad Prism software, and EC_50_s (effective concentrations that inhibit 50% of maximal growth) were derived from sigmoidal dose-response curves with variable slopes. The EC_50_s reported here are the averages of at least three independent experiments, except for DFMO (d,l-alpha-difluoromethylornithine [Eflornithine]), where *n* = 2.

### Quantification of intracellular cAMP concentration.

The intracellular concentration of cAMP in bloodstream form T. brucei brucei cell lines, upon incubation with various phosphodiesterase inhibitors, was measured as described previously (19) using the Direct Cyclic AMP Enzyme Immunoassay kit (Assay Designs). Samples were taken in duplicate, and all assays were conducted independently at least three times.

### PCR and sequencing of selected genes.

Clonal cultures of the parental wild-type T. brucei brucei Lister 427 strain and the CpdA-resistant R0.8 line were derived by limiting serial dilution, with that of the R0.8 cell line conducted under selective pressure of 0.4 μM CpdA; genomic DNA was extracted from each clonal cell line as described previously (31). The proofreading polymerase KOD (Novagen) was used to amplify the genes under standard reaction conditions. Once the cycles were completed, 1 U GoTaq DNA polymerase (Promega) was added to each reaction mixture and incubated at 72°C for 10 min to add adenine nucleotide overhangs to the amplification products. The amplicons were then separated by electrophoresis on a 1% (wt/vol) agarose gel, excised, gel purified, ligated into the pGEMT-easy vector (Promega), and used to transform Escherichia coli JM109 bacteria (Stratagene). Single bacterial colonies picked from selective agar plates were grown in 5 ml LB, after which the plasmid DNA was extracted and purified using a miniprep kit (Qiagen). BigDye Sanger sequencing (Eurofins-MWG-Operon) was carried out with T7 and SP6 primers and internal primers. Each of the four genes identified by the RNAi library screen (*CARP1* to -*4*) was also sequenced in the parental wild-type T. brucei brucei Lister 427 and R0.8 cell lines in a similar fashion. All primer sequences are available upon request.

### Genome-wide RNA interference screen for resistance to CpdA.

Detailed descriptions of the T. brucei brucei RNA library and approaches to screening have been published previously (32–34), and these methods were followed with only minor modifications. Briefly, a whole-genome RNAi library in bloodstream form T. brucei brucei strain Lister 427 was induced with 1 μg/ml Tet 24 h prior to the addition of 30 nM CpdA. While under CpdA selection, RNAi induction was maintained throughout; upon passage to fresh medium, the total number of cells transferred was never below 5 × 10^6^, in order to maintain library complexity. Growth was monitored daily by hemocytometer, and the cell density was adjusted as required with fresh medium containing CpdA and Tet. The inducibility of resistance to CpdA due to RNAi induction was assessed by monitoring growth for 72 h in the presence and absence of 1 μg/ml Tet and/or 60 nM CpdA. The RNAi target DNA fragments were amplified from the genomic DNA, sequenced, and identified as described previously (32).

### Western blot analysis.

Lysates of 4 × 10^6^ cells were separated on 10% polyacrylamide gels and blotted onto an Immobilon-FL polyvinylidene difluoride (PVDF) membrane (Millipore). Immunodetection of tagged CARP proteins was performed with anti-HA (mouse monoclonal antibody, clone 12CA5; kindly provided by E. Kremmer, Helmholtz Center Munich) or BB2 (Ty1 epitope [35]) antibodies in a 1:1,000 dilution. PFR-A/C detected by the monoclonal antibody L13D6 (dilution, 1:2,500 [36]) was used as an internal loading control. Infrared detection was performed using an IRDye 800CW goat anti-mouse IgG (H+L) secondary antibody (1:5,000) and the Odyssey IR fluorescence scanning system (both from LI-COR). Signals of tagged CARP proteins were normalized to the PFR-A/C loading control after automatic subtraction of the background values (median left/right method) using the Odyssey software (LI-COR).

### *CARP* gene transcript level analysis.

cDNA was generated by reverse transcription (iScript cDNA synthesis kit; Bio-Rad) of RNA isolated (NucleoSpin RNA II; Macherey-Nagel) from MiTat 1.2 Lister 427 or the derived CpdA-resistant R0.8 cell line treated or not with 0.1 μM CpdA for 2 h. Relative expression levels of *CARP* messenger RNAs were determined by quantitative real-time PCR (with the FAST SYBR green Master Mix from Applied Biosystems and the CFX96TM Real-Time PCR Detection system from Bio-Rad) using the following cycling parameters: [5 min at 95°C; 40 × (30 s at 95°C, 30 s at 60°C)]. *TERT* was used as the reference gene (37). The primer sequences are available on request.

## RESULTS

### Selection for resistance to CpdA.

**Fig 1:**
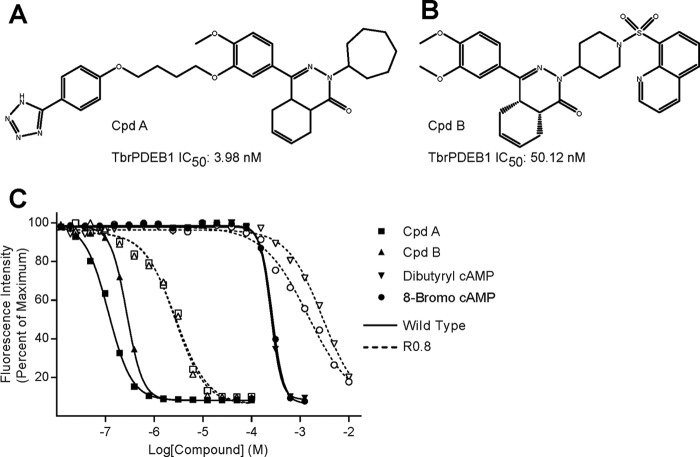
(A and B) Chemical structures of two novel tetrahydrophthalazinone PDE inhibitors, CpdA (19) and CpdB, with their IC_50_s against recombinant TbrPDEB1 (G. J. Sterk, personal communication). (C) Representative dose-response curves of trypanosome killing by both PDE inhibitors and two cell-permeable cAMP analogues assayed against wild-type bloodstream form T. brucei brucei Lister 427 strain (solid lines, filled symbols) and the CpdA-resistant R0.8 strain (dashed lines; unfilled symbols). See Table 1 for mean EC_50_s.

CpdA (Fig. 1A), a tetrahydrophthalazinone, has been demonstrated previously to be a highly potent inhibitor of cAMP-specific phosphodiesterase B (PDEB) enzymes in T. brucei brucei (19). Incubation with low concentrations of CpdA results in sustained elevation of intracellular cAMP, ultimately leading to cell death and validating PDEs as novel drug targets for potential chemotherapies against human African trypanosomiasis (HAT) as well as animal infections (19). In order to further dissect the mode of action of CpdA in T. brucei brucei, as well as to identify potential modes of resistance to tetrahydrophthalazinones, cells resistant to CpdA were selected. Bloodstream form trypanosomes were exposed to the chemical mutagen MMS to generate a heterogeneous mutated population. The culture was then exposed to a normally lethal concentration of CpdA (0.1 μM), and the surviving trypanosomes were continuously cultured in gradually increasing concentrations of the PDE inhibitor. After 2 months of culturing, the maximum tolerated concentration of CpdA was above 0.8 μM; a clonal cell line was obtained by limiting dilution and termed R0.8. The resistance phenotype was stable: it remained unaltered after 3 months of continuous culture in CpdA-free medium and also after storage in liquid nitrogen and subsequent thawing, as assessed by reexposure to 0.8 μM CpdA (data not shown).

### Resistance and cross-resistance characterization of the R0.8 cell line.

To more precisely quantify the degree of resistance to CpdA acquired by the R0.8 trypanosomes, *in vitro* efficacy assays were carried out. The EC_50_ for CpdA had increased >17-fold compared to the parental T. brucei brucei Lister 427 wild-type strain, from 0.08 ± 0.01 μM to 1.37 ± 0.19 μM (Fig. 1C; Table 1). Significant cross-resistance was displayed to another tetrahydrophthalazinone PDE inhibitor designated CpdB (Fig. 1B), showing a 9.7-fold increase in EC_50_ (Fig. 1C; Table 1). Conversely, no cross-resistance was observed with the mammalian PDE inhibitor dipyridamole (Table 1). However, the R0.8 cell line did display significant cross-resistance to the membrane-permeable cAMP analogues dibutyryl-cAMP and 8-bromo-cAMP, with 7.2- and 4.2-fold increases to their EC_50_s, respectively, compared to the parental Lister 427 strain (Fig. 1C; Table 1). Conversely, no significantly different sensitivity was observed for 8-(4-chlorophenylthio)-cAMP (8-CPT-cAMP) (Table 1). Nor did we observe any significant differences in the EC_50_s of the trypanocidal drugs used as controls, including the diamidines diminazene and pentamidine, the arsenicals cymelarsan and phenylarsine oxide, or to the nitroheterocycle nifurtimox. A slight but statistically significant increase in sensitivity to suramin was observed for the R0.8 cell line (Table 1).

**Table 1:** Resistance and cross-resistance characterization of the R0.8 bloodstream form cell line, compared to the parental wild-type T. brucei brucei strain Lister 427

Compound	Average EC_50_ (μM)		Resistance factor	*P* value^*a*^
	Lister 427	R0.8		
PDE inhibitors				
CpdA	0.08 ± 0.01	1.4 ± 0.2	17.2	0.004
CpdB	0.13 ± 0.03	1.28 ± 0.25	9.7	0.016
Dipyridamole	17.9 ± 2.7	9.2 ± 0.8	0.5	0.059
cAMP analogues				
Dibutyryl cAMP	263 ± 13	1890 ± 314	7.2	0.011
8-Bromo-cAMP	271 ± 8	1133 ± 185	4.2	0.014
8-(4-chlorophenylthio)-cAMP	1.24 ± 0.4	0.25 ± 0.05	0.2	0.201
Known trypanocides				
Suramin	0.0212 ± 0.0008	0.0156 ± 0.0005	0.7	0.001
Diminazene	0.022 ± 0.007	0.011 ± 0.001	0.5	0.133
Pentamidine	0.0016 ± 0.0004	0.0014 ± 0.0002	0.9	0.683
Cymelarsen	0.0038 ± 0.0004	0.0038 ± 0.0003	1.0	1.000
Phenylarsine oxide	0.00083 ± 0.00006	0.00088 ± 0.00011	1.1	0.783
Nifurtimox	2.01 ± 0.24	1.61 ± 0.08	0.8	0.246

a For comparison of R0.8 and Lister 427.

### Intracellular cAMP metabolism in the R0.8 strain.

**Fig 2:**
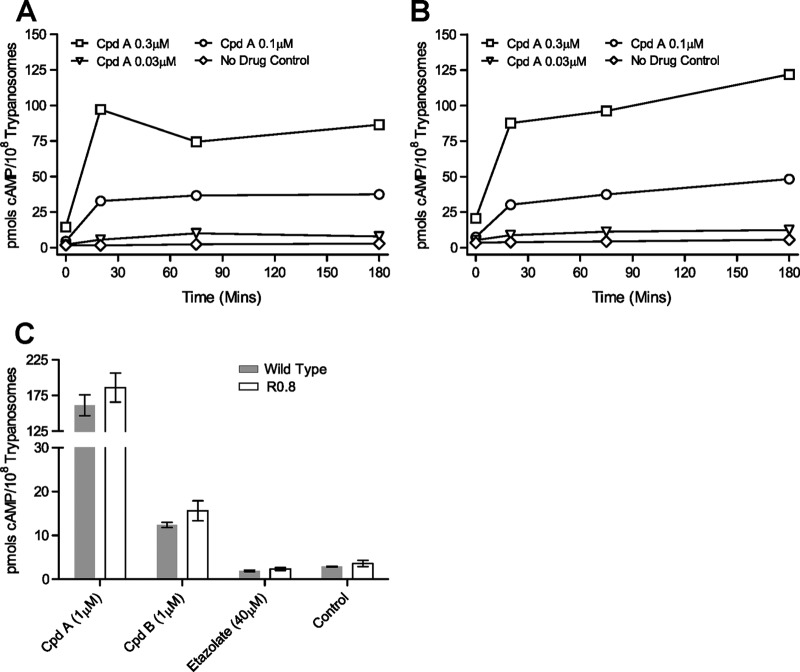
Intracellular cAMP concentrations elicited by CpdA in wild-type T. brucei brucei strain Lister 427 bloodstream form trypanosomes (A) and in the derived CpdA-resistant R0.8 strain (B); the graphs shown are representative of three paired, independent experiments. (C) Intracellular cAMP concentrations after incubation for 3 h with 1 μM CpdA, 1 μM CpdB, and 40 μM etazolate of Lister 427 wild-type bloodstream form trypanosomes (gray bars) or the derived CpdA-resistant R0.8 cell line (unfilled bars); error bars show standard errors of the means; *n* ≥ 3.

The intracellular concentration of cAMP was monitored over time on incubation with various concentrations of CpdA in the resistant R0.8 cell line and its parental T. brucei brucei wild-type strain Lister 427 (Fig. 2A and B). No significant difference (2-tailed, paired Student's *t* test) in the steady-state level of cAMP (i.e., the no-drug controls) was detected between the two cell lines over 3 h of observation. The addition of CpdA resulted in a rapid increase in the intracellular cAMP concentration within 20 min in both strains, and again no statistical differences between strains were observed at any of the CpdA concentrations used or at any of the time points sampled (Fig. 2A and B). CpdB also significantly raised the intracellular cAMP concentration compared to the no-drug control, with identical increases in both cell lines (Fig. 2C). The intracellular cAMP levels induced with CpdB are ∼10-fold lower than upon CpdA treatment at the same concentration, as expected from the >10-fold-lower affinity to target (the 50% infective concentration [IC_50_] for recombinant TbrPDEB is 3.98 nM for CpdA and 50.12 nM for CpdB; G. J. Sterk, personal communication). The mammalian PDE inhibitor etazolate had no effect on cAMP levels in both cell lines. The ORFs of both *TbrPDEB* genes were cloned from R0.8 and wild-type cells and sequenced, including the predicted untranslated regions (UTR). For *TbrPDEB1*, the wild-type parental strain contained two distinct alleles, with polymorphisms at positions 738, 1362, and 1602 of the ORF (see Fig. S1 in the supplemental material). The R0.8 strain appears to be homozygous, with all 9 plasmid clones having a sequence identical to that of allele B of the wild type. Twenty-eight allelic polymorphisms were identified in the ORF of *TbrPDEB2*, of which 27 are located in four clusters in the GAF-A domain (38) and 1 in the catalytic domain (base pair 2365; see Fig. S1 in the supplemental material) resulting in an amino acid change (codon 789; Cys in allele A and Ser in allele B). Both alleles were present in the R0.8 line; however, only the cysteine residue was present in each at codon 789. Thus, while some allelic recombination events appear to have occurred in the R0.8 cell line, no polymorphisms of either *TbrPDEB* gene were identified that were present only in the R0.8 strain. This is consistent with the unchanged basal and PDE inhibitor-induced cAMP concentrations in the R0.8 strain.

### An RNAi screen identifies genes involved in sensitivity to CpdA.

**Fig 3:**
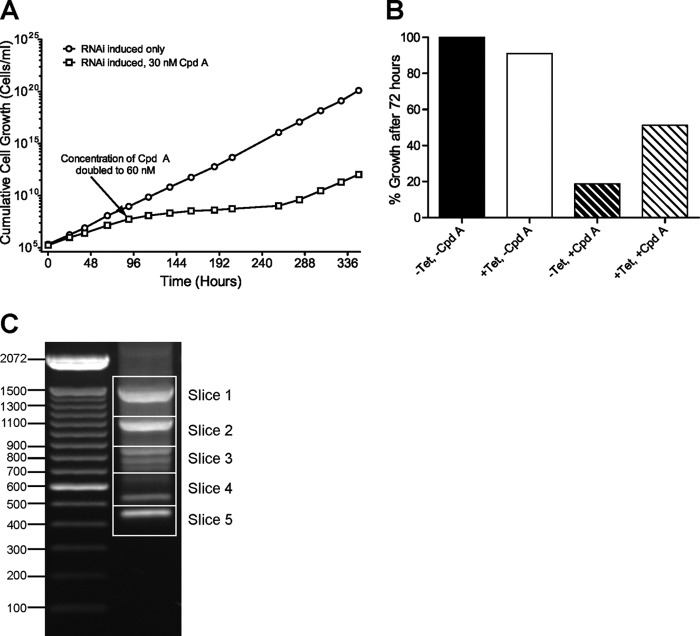
(A) Cumulative growth of an induced (1 μg/ml Tet) whole-genome RNAi library in bloodstream form T. brucei brucei Lister 427 strain trypanosomes in the presence (squares) or absence (circles) of CpdA. The initial concentration of CpdA was 30 nM, which was increased to 60 nM after 4 days. (B) Relative growth of the surviving RNAi library trypanosome population after selection with CpdA. Cells were grown for 72 h in the presence of 60 nM (+CpdA) or absence of (−CpdA) CpdA with RNAi either induced (+Tet) or uninduced (−Tet). Growth is expressed as a percentage of that of the −Tet, −CpdA population. (C) Ethidium bromide-stained agarose gel (1%, wt/vol) of the genomic PCR products representing the RNAi target fragments in the library constructs selected after 15 days in CpdA (i.e., those fragments that are associated with resistance to CpdA). DNA ladder size markers on the left are denoted in base pairs. Slices refer to the portions of the gel excised for cloning and sequencing.

In order to identify genes for cAMP effector proteins (e.g., components of a signaling cascade) rather than cAMP metabolism, that confer sensitivity to CpdA, a whole-genome RNAi screen was carried out. The bloodstream form RNAi library generated and described previously (32–34) was induced with tetracycline (Tet) for 24 h before selection with 30 nM CpdA. Four days of selection resulted in only a slight decrease in the growth rate of the CpdA-exposed Tet-induced culture, compared to the Tet-induced control without CpdA (Fig. 3A). Therefore, the selective concentration was increased to 60 nM CpdA. Subsequently, the population doubling time increased to over 24 h between days 5 and 11 and later returned to around 8 h (similar to control). Fifteen days after the initial selection with CpdA, genomic DNA was extracted from the +Tet/+CpdA culture of surviving trypanosomes for PCR cloning of RNAi target fragments. At the same time point, the effect of RNAi induction on population resistance to CpdA was analyzed (Fig. 3B). After 72 h of growth in fresh medium without Tet, cell density in the culture treated with 60 nM CpdA (−Tet/+CpdA) was 19% of that of the untreated culture (−Tet/−CpdA). In Tet-induced cultures, growth in the presence of 60 nM CpdA (+Tet/+CpdA) was 56% of that of the untreated control. Thus, resistance to CpdA in the selected population is, at least in part, due to induction of RNAi.

PCR amplification of the RNAi target fragments from the resistant population gave several products, comprising at least eight discrete visible bands following gel electrophoresis (Fig. 3C). Five contiguous regions of the gel were excised, and the DNA was purified and cloned in E. coli. Multiple clones from each excised region, representing all the different RNAi target fragment sizes, were sequenced and mapped to the reference genome (39) using TriTrypDB (40). Ten distinct RNAi target fragments were obtained from the 24 clones sequenced, representing all eight bands in the agarose gel (Table 2 and Fig. 3C). Three ORFs were identified by multiple, independent RNAi target fragments, and one was identified by a single RNAi fragment; the genes were designated *CARP1* to *CARP4* for cAMP Response Protein 1 to 4, and their identifications are listed in Table 2.

**Table 2:** Systematic gene IDs of RNAi target fragments selected with CpdA

Gene name	Gene ID		Length (no. of amino acids)	RNAi target fragment(s)	
	Strain TREU 927	Strain Lister 427		No.	Size(s) (bp)
*CARP1*	Tb927.11.16210	Tb427tmp.01.7890	705	3	446, 851, 1,101
*CARP2*	Tb927.11.12860	Tb427tmp.52.0004	302	2	736, 1,507
*CARP3*	Tb927.7.5340	Tb427.07.5340	498	4	386, 431, 532, 635
*CARP4*	Tb927.3.1040/60	Tb427.03.1040/60	779	1	780

**Fig 4:**
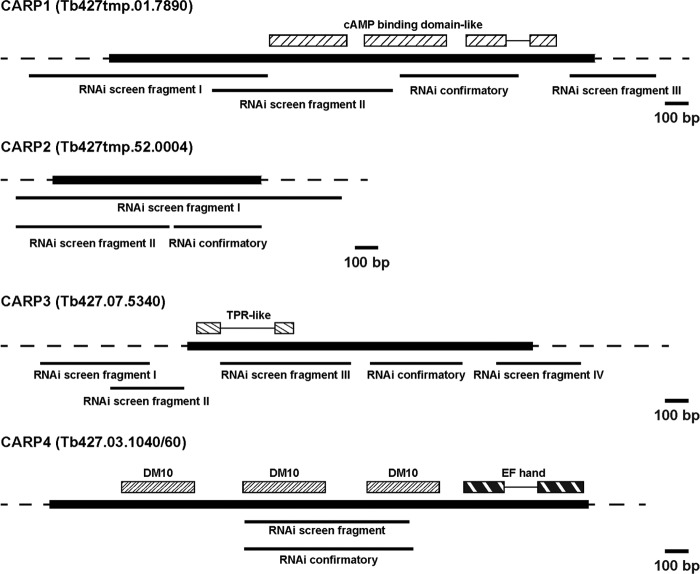
Maps of the genomic loci of the *CARP* genes, RNAi target fragments, and domain annotations. The sequence data are from tritrypdb.org; ORFs are indicated in black. “RNAi screen fragments” were identified as described for Fig. 3C; “RNAi confirmatory fragments” are the target fragments designed for the experiments shown in Fig. 5 and 6. Domain architecture was analyzed using Smart (smart.embl-heidelberg.de/) and Superfamily (supfam.cs.bris.ac.uk). cAMP binding-domain-like, SSF51206; DM10, SM000676; EF hand, SSF47473; TPR-like, SCOP48452. Bar, 100 bp.

One of the genes knocked down in the CpdA-resistant cultures was Tb427tmp.01.7890 (*CARP1*; Tb927.11.16210 in T. brucei brucei reference strain TREU 927), encoding a 705-amino-acid protein containing two apparently intact and one partial cyclic AMP binding-like domains (Fig. 4) that is conserved in synteny in each of the kinetoplastid genomes sequenced. No close orthologues were identified in other organisms, but cyclic nucleotide-dependent kinases and ion channels appear to be the most closely related proteins outside the Kinetoplastida.

**Fig 5:**
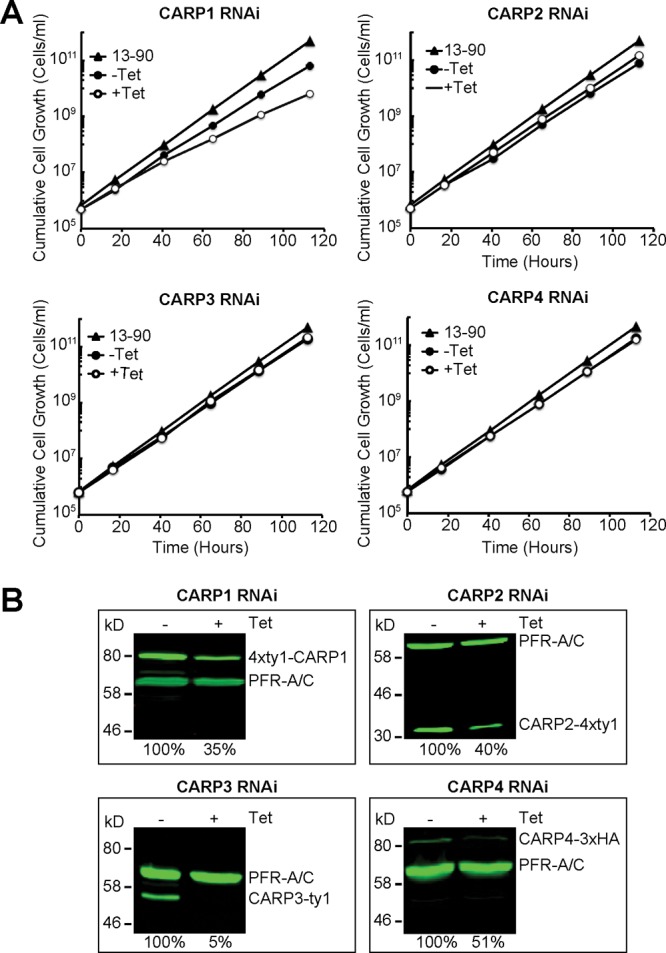
Independent RNAi targeting of identified *CARP* genes. (A) Cumulative growth of *CARP* RNAi cell lines in the presence (+Tet, empty circles) or absence (−Tet, filled circles) of 1 μg/ml tetracycline. The parental 13-90 cell line was included as a control (filled triangles). The cells were counted and diluted daily in order to keep the cell density below 8 × 10^5^/ml. (B) Western blot analysis of CARP protein expression in the presence (+Tet, 24 h) or absence (−Tet) of 1 μg/ml tetracycline. CARP1 was tagged at the N terminus with a 4×Ty1 tag, CARP2 with a C-terminal 4×Ty1 tag, CARP3 with a C-terminal Ty1 tag, and CARP4 with a C-terminal 3×HA tag. CARP protein levels were normalized to PFR-A/C detected by the monoclonal antibody L13D6 (36) and set to 100% in the absence of Tet. The relative scan gain in the 800-nm channel was set to 1 for the CARP1 Western blot and to 3 for the CARP2 to -4 Western blots. Relative expression levels are indicated as percentages of expression in the noninduced cultures.

*CARP2* (Tb427tmp.52.0004; Tb927.11.12860 in TREU 927) codes for a hypothetical protein of 302 amino acids, but a downstream alternative start codon may produce a shorter protein of 235 amino acids (41). This corresponds to the ORF length of the majority of *CARP2* homologues that are well conserved across the Kinetoplastida (>82% amino acid identity in all Trypanosoma spp. and >59% identity in Leishmania spp.) and many other species, including humans (47.7% identity). The apparent molecular mass of the C-terminally tagged T. brucei brucei protein (see Western blot in Fig. 5B) shows that the first ATG is in fact used and that the trypanosomal CARP2 carries an N-terminal extension. There is no known function, and no recognizable functional domains could be identified in any of the homologues. It has been detected in proteomes of T. brucei brucei flagellum (42) and of cytoskeletal and plasma membrane fractions (43), as well as in an *in silico* predicted proteome of the flagellar and basal body of Chlamydomonas reinhardtii (44, 45).

*CARP3* (Tb427.07.5340; Tb927.7.5340 in TREU 927) encodes a hypothetical protein of 498 amino acids with orthologues only in Trypanosoma spp. and strains. A BLASTP search identified the putative stibogluconate resistance gene family in Leishmania spp. as the closest homologue outside trypanosomes (Leishmania braziliensis LBRM_31_1110; 20.4% identity); amplification of this gene family in Leishmania tarentolae resulted in resistance to antimony-containing drugs (46). The protein was found in the plasma membrane-enriched fractions of bloodstream T. brucei brucei (43) and in mitochondrial fractions of procyclic trypanosomes (47) and is possibly palmitoylated (48). At the N-terminal end of the protein, a weak TPR-like domain (tetratricopeptide repeat) signature is detected. TPR domains can mediate protein-protein interactions such as dimerization and the assembly of multiprotein complexes (49).

The fourth ORF identified from the RNAi target fragments, *CARP4* (Tb927.3.1040/60), is a hypothetical gene that spans three automatically annotated ORFs in release 5.0 of TriTrypDB (T. brucei brucei TREU 927 strain; the respective sequence segment of strain Lister 427 is annotated as incomplete). However, the middle ORF appears to be a sequence contaminant disrupting a single open reading frame encompassing Tb927.3.1040 and Tb927.3.1060. The middle ORF is absent from all RNA sequencing (RNAseq) data on the TriTrypDB website and has no homologues or orthologues in any of the other kinetoplastid genomes on the database. The full-length Tb927.3.1040/60 ORF, on the other hand, is conserved in synteny in all kinetoplastid genomes sequenced to date, with amino acid identity of 53.6% in Leishmania major and 96.3% in T. brucei gambiense. The combined Tb927.3.1040/60 ORF codes for a hypothetical protein of 779 amino acids and is predicted to have three DM10 domains and one EF-hand domain located at the C-terminal end (Fig. 4). BLASTP and domain architecture (NCBI CDART) searches uncovered three other genes in T. brucei brucei strain 927 containing the same domain architecture (Tb927.11.1430, Tb927.5.2950, and Tb927.10.7690).

### All four *CARP* genes confer sensitivity to CpdA.

Independent RNAi constructs individually targeting each of the four genes identified by the RNAi screen were generated and transfected into the T. brucei brucei Lister 427 strain MiTat 1.2 13-90 cell line for tetracycline-inducible expression. When possible, specific RNAi target sequences that do not overlap the target sequences returned from the RNAi screen were chosen (Fig. 4). For *CARP4*, a target fragment covering the central part of the combined ORF Tb927.3.1040/60 was amplified from Lister 427 genomic DNA, sequenced, and cloned into the RNAi vector (25). This provided proof of a contiguous ORF in strain Lister 427 and a possible sequence assembly error and misannotation in that region of the reference TREU 927 genome sequence. Growth of the parental and transfected uninduced or induced (1 μg/ml tetracycline) trypanosomes was monitored over 120 h (Fig. 5A). *CARP1* RNAi resulted in a slight growth phenotype, which was noticeable in part without tetracycline induction, probably the result of “leaky” RNAi repression.

**Fig 6:**
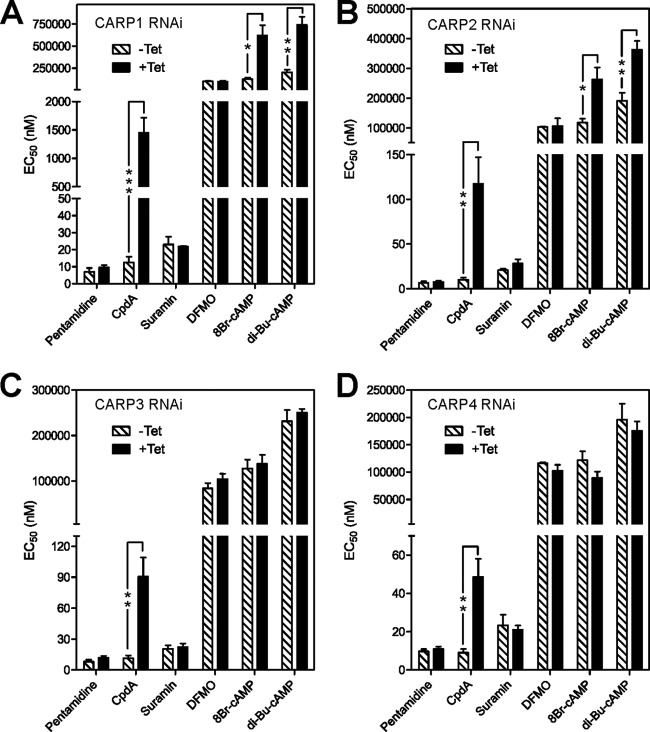
Validation of identified *CARP* genes for function in CpdA susceptibility. EC_50_s as determined by alamarBlue assay are presented as means of three or more independent determinations. Uninduced EC_50_s (hatched bars) were determined in parallel with induced (1 μg/ml Tet for 24 h, solid bars) EC_50_s. The significance of differences between uninduced and induced was tested by a paired two-tailed Student's *t* test as indicated: *, *P* < 005; **, *P* < 0.02; ***, *P* < 0.01.

To quantify the RNAi-mediated knockdown of CARP protein amounts, each *CARP* gene was tagged *in situ* in the respective RNAi clone for quantitative Western blot analysis of endogenous expression levels (Fig. 5B). RNAi induction for 24 h caused a substantial reduction of the specific tagged CARP protein (Fig. 5B). The strongest repression was observed for CARP3 (to 5%), whereas only a 2- to 3-fold reduction of CARP1, CARP2, or CARP4 protein levels was detected. For CARP1, reliability of the quantification was confirmed by several independent cell lines *in situ* tagged at the N or C terminus using a 4×Ty1 or 3×HA tag, respectively (see Fig. S2 in the supplemental material). For selected clones (the ones shown in Fig. 5A) the EC_50_ for CpdA was determined by the alamarBlue cell viability assay with and without induction of RNAi. As controls, several trypanocidal drugs in use for therapy were included. No cross-resistance to pentamidine, suramin, or DFMO (eflornithine) was observed for any of the clones upon *CARP* RNAi induction. In contrast, RNAi-mediated knockdown of all *CARP* genes conferred significant resistance to CpdA (Fig. 6). The degree of resistance to CpdA was highest upon knockdown of *CARP1* (117-fold; *P* < 0.01) and was 10.1-fold, 7.9-fold, and 5.4-fold for knockdown of *CARP2*, *CARP3*, and *CARP4*, respectively. The effect of the RNAi knockdown on sensitivity to lipophilic cAMP analogues was also investigated. *CARP1* knockdown resulted in 5.0- and 3.7-fold increases of the EC_50_ for 8-bromo-cAMP and dibutyryl-cAMP, respectively. Similarly, *CARP2* knockdown also resulted in resistance to 8-bromo-cAMP and dibutyryl-cAMP, but to the lesser extents of 2.2- and 1.9-fold, respectively. For *CARP3* and *CARP4* the differences were not significant.

### Sequencing and transcript levels of candidate resistance genes in the R0.8 cell line.

Each of the four *CARP* genes identified by the RNAi screen was PCR amplified from the CpdA-resistant R0.8 cell line, cloned, and sequenced for mutations in the ORF, as well as in any predicted UTR regions. Multiple clones for each gene were sequenced and aligned; however, no polymorphisms could be identified in the R0.8 strain that were not present in at least one allele of the parental T. brucei brucei Lister 427 wild-type strain. Similarly, quantitative PCR (qPCR) data comparing transcripts of each of the four *CARP* genes in the wild type versus the R0.8 cell line showed no difference in transcript abundance, either in the presence or absence of CpdA (see Fig. S3 in the supplemental material). In conclusion, the CpdA resistance of the R0.8 line cannot be attributed to mutations in the identified *CARP* genes or to reduced *CARP* transcript levels. Although protein expression remains to be investigated, it seems likely that additional genes are involved in resistance of the R0.8 line to elevated cAMP.

## DISCUSSION

In order to exploit the full therapeutic potential of PDE inhibitors in the future, an understanding of how resistance, if any, might arise in the field is essential. Moreover, a full understanding of the action of any PDE-targeting trypanocides is hampered by the almost complete absence of information about intracellular cAMP signaling in T. brucei brucei and related kinetoplastids, beyond characterization of families of adenylyl cyclases (ACs) and PDEs (17). Two approaches were employed to investigate potential modes of resistance: (i) mutagenesis and selection of cell lines resistant to the trypanosomal PDE inhibitor CpdA, followed by their characterization; and (ii) a whole-genome RNAi screen for drug efficacy determinants of CpdA.

A substantial level of resistance to CpdA was induced in T. brucei brucei, resulting in the R0.8 cell line. Resistance to CpdA conferred cross-resistance to another tetrahydrophthalazinone PDE inhibitor, CpdB, identified in the same high-throughput screen with recombinant TbrPDEB. Not surprisingly, resistance to one PDE inhibitor gives resistance to the entire inhibitor class; indeed, two additional related tetrahydrophthalazinone compounds also showed similar cross-resistance profiles (data not shown). On exposure to tetrahydrophthalazinones, the cAMP level in the wild-type and resistant R0.8 populations increase similarly, indicating that resistance is not caused by mutated PDEs or adapted PDE expression. Thus, in the R0.8 cell line, resistance must be based on tolerating high intracellular cAMP. This is also compatible with the cross-resistance observed for the cAMP analogues dibutyryl cAMP and 8-bromo cAMP and consistent with the lack of mutations in the *PDEB* gene sequences in the R0.8 trypanosomes. The absence of an effect of etazolate on cAMP levels in T. brucei brucei shows that this compound, previously reported to inhibit T. brucei brucei PDEB1 (50), does not, in fact, act as an effective PDE inhibitor on T. brucei brucei cells.

Given that CpdA is quite lipophilic, it is expected to diffuse rather than be transported across the plasma membrane, so that uptake-related resistance is not possible, in contrast to actively accumulated trypanocidal drug classes like the diamidines (51). Importantly, no cross-resistance was observed with the current trypanosomiasis drugs, including diamidines, arsenicals, suramin, and nifurtimox, showing that PDE inhibitors have a distinct mechanism of resistance. Thus, combinations with current drugs could significantly delay the onset of treatment failures and/or improve the effectiveness of the currently unsatisfactory armamentarium against HAT.

Surprisingly, the R0.8 line was not resistant to the cAMP analogue 8-CPT-cAMP, which is widely used as a cAMP agonist in mammalian cells and induces cell cycle arrest and stumpy stage development in T. brucei brucei (52). However, it has been shown that products of intracellular hydrolysis of 8-CPT-cAMP are responsible for growth inhibition, by a cAMP-independent mechanism (53). The observed lack of cross-resistance to 8-CPT-cAMP corroborates this. This analogue does not qualify as a cAMP agonist in trypanosomes, and hence the lack of cross-resistance is compatible with CpdA resistance resulting from changes in cAMP effector proteins.

RNAi library screening has proven to be a powerful approach for uncovering novel genes involved in the mode of action of many of the current trypanocides (11, 32, 34) and, consequently, candidates for changes associated with resistance. While the specific target of CpdA is the PDEB family of proteins (19), the targets of the resulting increase in cAMP were unknown. In this study, RNAi library screening uncovered four putative cAMP target or effector proteins. Although cAMP metabolism has been validated as a drug target in trypanosomes (18, 19) and the signaling molecule has important roles in cell division and cytokinesis (19, 21), this is the first time that cAMP response proteins have been identified in this pathogen, showing the power of this genomic approach.

Knockdown by RNAi of *CARP1* resulted in >100-fold increases in EC_50_ for CpdA. The prediction of cyclic nucleotide binding-like domains in CARP1 is clearly suggestive of a pivotal part to play in the cAMP signaling cascade by this protein, although cAMP binding will need to be experimentally verified. This is particularly significant, as all the cAMP effectors widely conserved among other organisms either have no detectable orthologues in the T. brucei brucei genome (EPAC and cNMP-gated ion channels) or are refractory to cAMP and have acquired a distinct mode of regulation (protein kinase A [PKA]-like kinase [54]; S. Bachmaier and M. Boshart, unpublished data). CARP1 may thus be part of the first second-messenger signaling cascade to be delineated in kinetoplastids. We propose that the CARP2 to -4 proteins, whose repression resulted in more-moderate but still highly significant CpdA resistance, are likely to be part of the same signaling pathway as CARP1 or even associated in a complex. CARP2 and CARP4 are both predicted as conserved proteins in motile flagella of several organisms, along with the three other 3× DM10 domain-containing proteins similar to CARP4 (55). This may link to the cytokinesis phenotype resulting from aberrant cAMP levels (19, 21), since a crucial role for the trypanosome flagellum in cytokinesis is well documented (56). The localization of TbrPDEB1 and B2 (18) and adenylate cyclases (57) to the flagellum is consistent with this hypothesis. For CARP2, we provide the first functional assignment for this highly conserved eukaryotic flagellar protein of previously unknown function. Interestingly, a human homologue of CARP4, EFHC1, has been shown to be a component of axonemes and cilia, with mutations in EFHC1 being implicated in juvenile myoclonic epilepsy (58, 59). This suggests that T. brucei brucei may be an exciting model organism to further investigate the functions of these critical, but poorly characterized, DM10 domain-containing proteins.

In summary, resistance to PDE inhibitors by bloodstream form T. brucei brucei can occur and has been found downstream of the PDEs in the cAMP signaling cascade, which is currently undefined in trypanosomes. However, four potential downstream cAMP effector proteins are already reported here, and reduced expression of any one of them by RNAi results in resistance to PDE inhibitors. While much work needs to be done to fully characterize these proteins, they could potentially be the first *bona fide* downstream cAMP effector proteins identified in Trypanosoma brucei and provide the first step to mapping the downstream cAMP signaling cascade. As no mutations or changes in transcript level in any of the four *CARP* genes could be detected in the resistant R0.8 cell line, analysis of such lines may reveal additional components of that pathway in the future. Finally, CARP1 may be a good drug target in its own right, as it is specific to kinetoplastid parasites and appears to have cyclic nucleotide binding-like pockets. The huge experience of the pharmaceutical industry in designing inhibitors and activators for cNMP-binding proteins would be a distinct advantage in this case.

## Supplementary Material

Supplemental material
