# Supplementary material for: Cyclic AMP Effectors in African Trypanosomes Revealed by Genome-Scale RNA Interference Library Screening for Resistance to the Phosphodiesterase Inhibitor CpdA
Source: Antimicrob Agents Chemother. 2013 Oct;57(10):4882–93. doi: 10.1128/AAC.00508-13 (PMC3811416; doi:10.1128/AAC.00508-13)
Supplement: Supplemental material [file supp_57_10_4882__index.html]

Supplemental material 

# Cyclic AMP Effectors in African Trypanosomes Revealed by Genome-Scale RNA Interference Library Screening for Resistance to the Phosphodiesterase Inhibitor CpdA

## Supplemental material

**Files in this Data Supplement:**

- Supplemental file 1 -

  Supplemental Figures S1 to S3.

  PDF, 893K
