# Supplementary material for: Cyclic AMP Effectors in African Trypanosomes Revealed by Genome-Scale RNA Interference Library Screening for Resistance to the Phosphodiesterase Inhibitor CpdA
Source: Antimicrob Agents Chemother. 2013 Oct;57(10):4882–93. doi: 10.1128/AAC.00508-13 (PMC3811416; doi:10.1128/AAC.00508-13)
Supplement: Supplemental material [file AAC.00508-13_zac010132207so1.pdf]

FIG. S1. Schematic diagrams of *TbrPDEB1* and *TbrPDEB2* showing functional domains (grey boxes) and allelic polymorphisms in *T. b. brucei* strain Lister 427 and the Cpd A resistant R0.8 cell line. The ORFs are represented by the largest box (white and grey parts); flanking regions are represented by thick black horizontal lines. Allelic polymorphisms are represented by thin (single nucleotide) or thicker (clusters of multiple nucleotides) vertical lines.

FIG. S2. Western Blot analysis of CARP1 repression (1 µg/ml tetracycline for 24h, + Tet) in additional *CARP1* RNAi cell lines with *in situ* tagged CARP1 either with an N-terminal 4xTy1 (upper panel) or a C-terminal 3xHA tag (lower panel). CARP1 protein levels were normalized to PFR-A/C detected by the monoclonal antibody L13D6 (36) and set to 100% in the absence of Tet. The relative scan gain in the 800 nm channel was set to 1 for the upper panel and to 3 for the lower panel. Relative expression levels are indicated as percentage of the non-induced cultures.

FIG. S3. *CARP1-4* mRNA expression levels in *T. b. brucei* strain Lister 427 wild type (WT) and derived R0.8 cell line in the presence or absence of Cpd A, determined by quantitative real-time PCR. Relative expression normalized to *TERT* as reference gene (37) and the wild type line is given with standard error (SEM) of three biological replicates.

S1

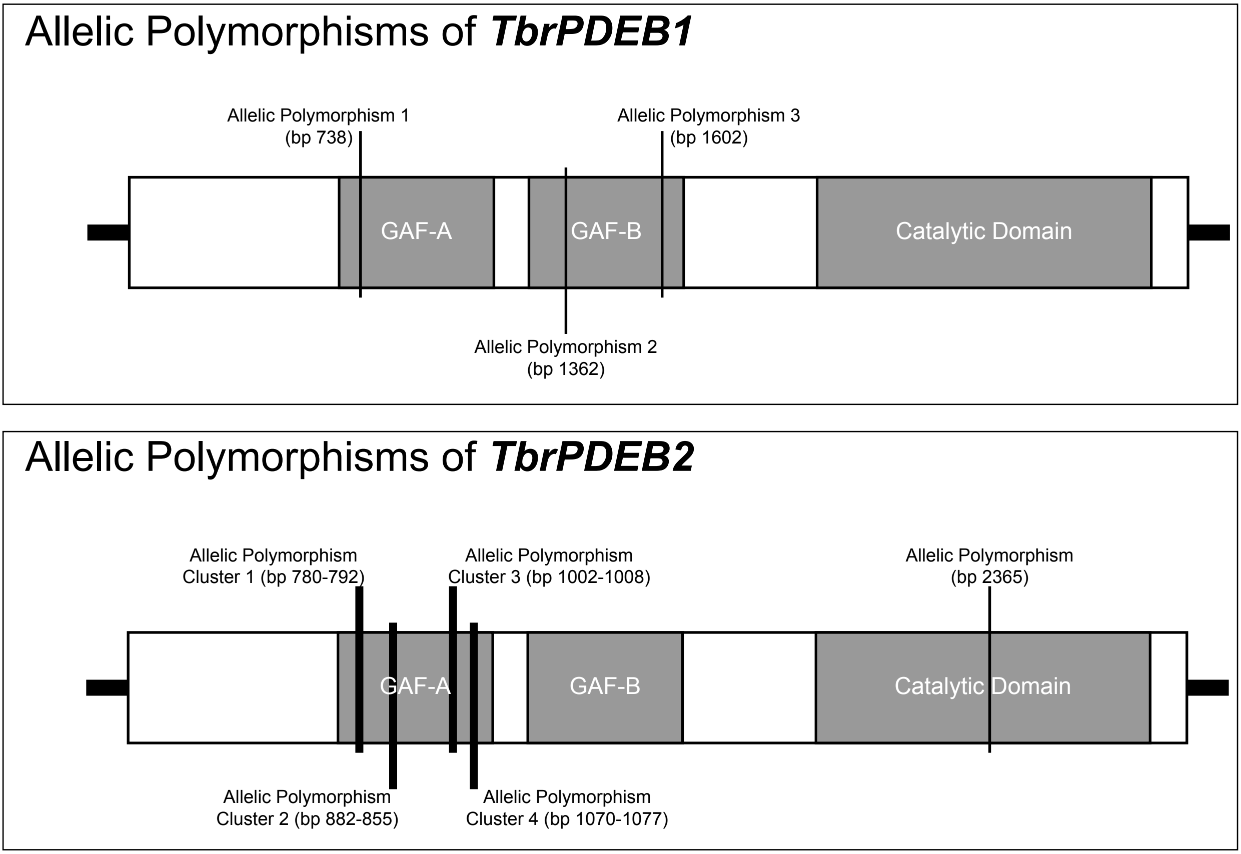
Allelic Polymorphisms of TbrPDEB1
Allelic Polymorphism 1
(bp 738)
Allelic Polymorphism 3
(bp 1602)
GAF-A
GAF-B
Catalytic Domain
Allelic Polymorphism 2
(bp 1362)
Allelic Polymorphisms of TbrPDEB2
Allelic Polymorphism
Cluster 1 (bp 780-792)
Allelic Polymorphism
Cluster 3 (bp 1002-1008)
Allelic Polymorphism
(bp 2365)
GAF-A
GAF-B
Catalytic Domain
Allelic Polymorphism
Cluster 2 (bp 882-855)
Allelic Polymorphism
Cluster 4 (bp 1070-1077)

S2

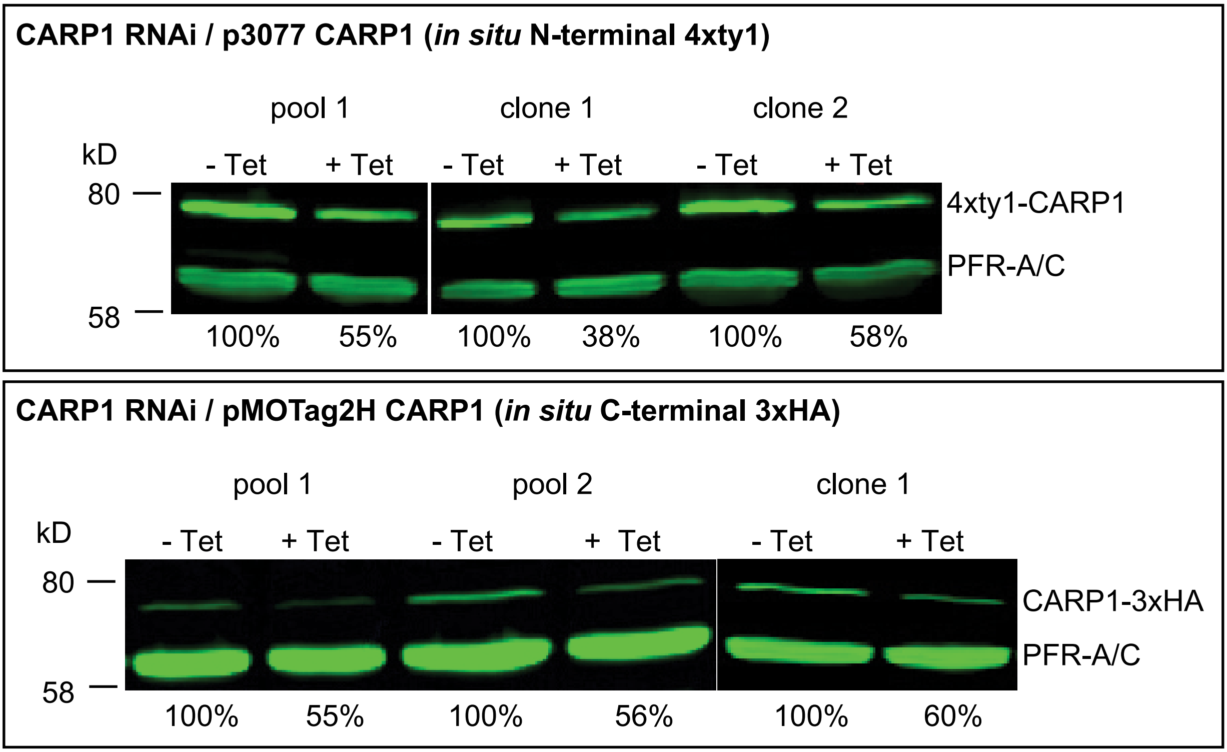
CARP1 RNAi / p3077 CARP1 (in situ N-terminal 4xty1)
pool 1
clone 1
clone 2
kD
- Tet
+ Tet
- Tet
+ Tet
- Tet
+ Tet
80
58
4xty1-CARP1
PFR-A/C
100%
55%
100%
38%
100%
58%
CARP1 RNAi / pMOTag2H CARP1 (in situ C-terminal 3xHA)
pool 1
pool 2
clone 1
kD
- Tet
+ Tet
- Tet
+ Tet
- Tet
+ Tet
80
58
CARP1-3xHA
PFR-A/C
100%
55%
100%
56%
100%
60%

S3

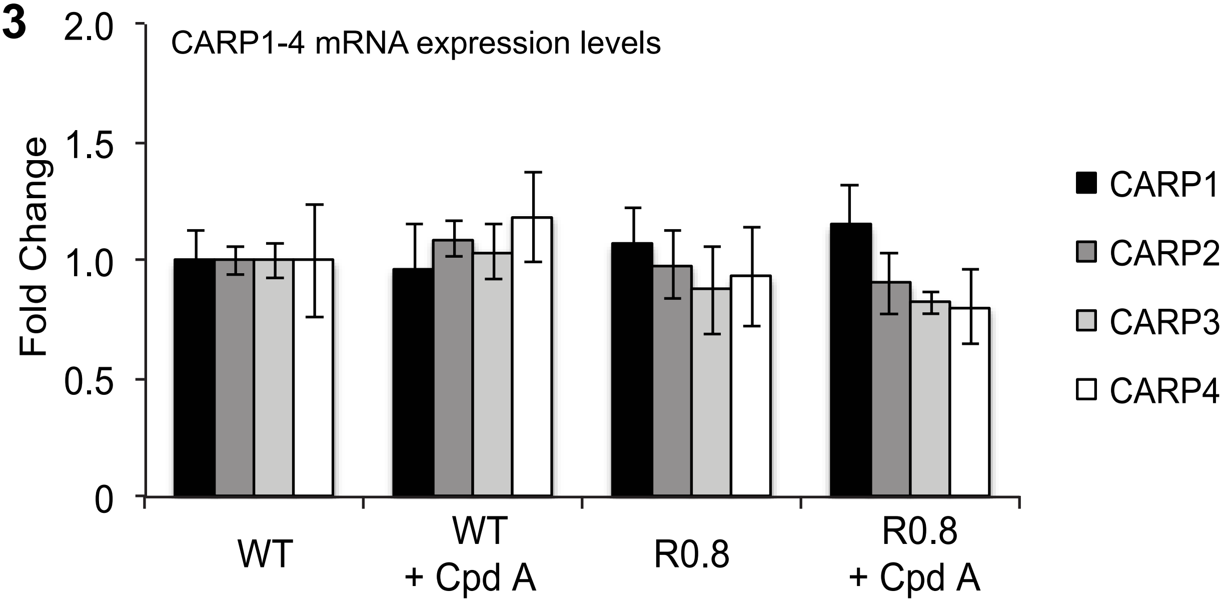

CARP1-4 mRNA expression levels
Fold Change
2.0
1.5
1.0
0.5
0
WT
WT + Cpd A
R0.8
R0.8 + Cpd A
CARP1
CARP2
CARP3
CARP4
